# *Archaeoglobus Fulgidus* DNA Polymerase D: A Zinc-Binding Protein Inhibited by Hypoxanthine and Uracil

**DOI:** 10.1016/j.jmb.2016.06.008

**Published:** 2016-07-17

**Authors:** Javier Abellón-Ruiz, Kevin J. Waldron, Bernard A. Connolly

**Affiliations:** Institute for Cell and Molecular Biology, University of Newcastle, Newcastle upon Tyne NE2 4HH, UK

**Keywords:** Pol-B, family-B DNA polymerase, Pol-D, family-D DNA polymerase, DP1, small (proofreading exonuclease) subunit of DNA polymerase D, DP2, large (polymerase) subunit of DNA polymerase D, Afu, *Archaeoglobus fulgidus*, Pfu, *Pyrococcus furiosus*, ICP-MS, inductively coupled plasma mass spectroscopy, Archaea, DNA polymerase D, uracil, hypoxanthine, Zn-binding protein

## Abstract

Archaeal family-D DNA polymerases (Pol-D) comprise a small (DP1) proofreading subunit and a large (DP2) polymerase subunit. Pol-D is one of the least studied polymerase families, and this publication investigates the enzyme from *Archaeoglobus fulgidus* (Afu Pol-D). The C-terminal region of DP2 contains two conserved cysteine clusters, and their roles are investigated using site-directed mutagenesis. The cluster nearest the C terminus is essential for polymerase activity, and the cysteines are shown to serve as ligands for a single, critical Zn^2 +^ ion. The cysteines farthest from the C terminal were not required for activity, and a role for these amino acids has yet to be defined. Additionally, it is shown that Afu Pol-D activity is slowed by the template strand hypoxanthine, extending previous results that demonstrated inhibition by uracil. Hypoxanthine was a weaker inhibitor than uracil. Investigations with isolated DP2, which has a measurable polymerase activity, localised the deaminated base binding site to this subunit. Uracil and hypoxanthine slowed Afu Pol-D “in *trans*”, that is, a copied DNA strand could be inhibited by a deaminated base in the alternate strand of a replication fork. The error rate of Afu Pol-D, measured *in vitro*, was 0.24 × 10^− 5^, typical for a polymerase that has been proposed to carry out genome replication in the Archaea. Deleting the 3′–5′ proofreading exonuclease activity reduced fidelity twofold. The results presented in this publication considerably increase our knowledge of Pol-D.

## Highlights

• Archaeal DNA Polymerase-D binds Zn, this is essential for activity.
• Archaeal DNA Polymerase-D is inhibited by uracil and hypoxanthine.
• Inhibition by deaminated bases occurs both in *cis* and in *trans.*
• Deaminated bases are sensed by the large (polymerase) subunit.
• Polymerase-D has high fidelity, compatible with genome replication.

## Graphical Abstract

**Figure:**
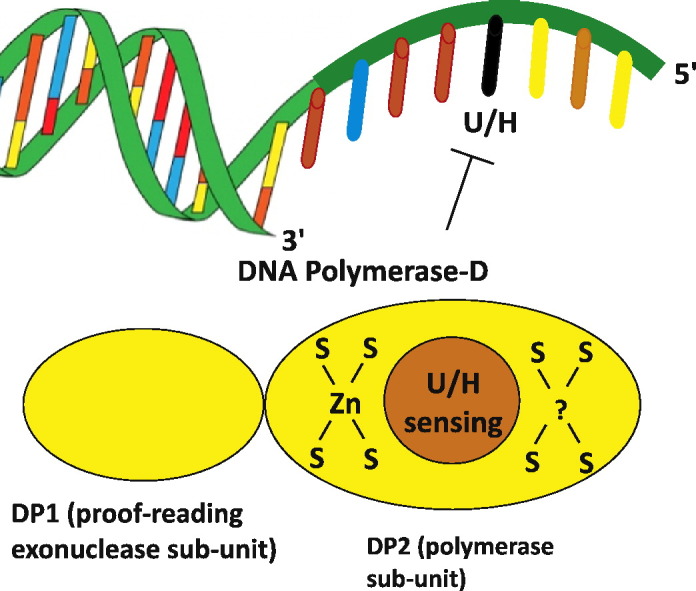

Accurate transmission of the data encoded within the genome requires faithful replication, a function performed by the replisome, a multiprotein complex of which DNA polymerases form a key component [1], [2], [3], [4], [5], [6], [7]. Based on the amino acid sequence similarity and structural information, DNA polymerases are classified into seven families: A, B, C, D, E, X, and Y; a subset of which, mainly from families A, B, C, and D, is dedicated to replication [1], [2], [3]. In Bacteria, it was historically thought that two molecules of the family-C DNA polymerase III, arranged in an asymmetric fashion, copied the leading and lagging strand [5]. More recently, a different arrangement has been suggested, requiring three copies of DNA polymerase III; one associated with leading strand replication and two with the lagging strand [6]. With Eukaryotes, it is clear that the two different family-B enzymes, polymerases δ and ε, are responsible for replication [7]. Genetic experiments, using *Saccharomyces cerevisiae*, initially implicated Pol δ in lagging strand copying and Pol ε in leading [8], [9]. More recently, using similar techniques in yeast strains of different genetic backgrounds, it has been suggested that Pol δ is responsible for replicating both DNA strands [10]. Information available for the Archaea is not as complete as for Bacteria or Eukaryotes. All archaeal lineages contain at least one family-B DNA polymerase (Pol-B), and in some phyla, such as the Crenarchaeota, more than one member is present [2], [11], [12]. Additionally, Archaea, with the notable exception of the Crenarchaeota, possess a family-D polymerase (Pol-D), unique to this domain [13], [14], [15], [16]. Traditionally, it has been assumed that archaeal replication is performed by a family-B polymerase. Support for this assertion arises from the observation that the archaeal replication machinery is similar to the eukaryotic system, coupled with the clear use of family-B enzymes for copying DNA in Eukaryotes [2], [8], [9], [10], [11], [12]. Furthermore, in the Crenarchaea, the family-B polymerase is the only enzyme present with features compatible with DNA replication [2], [11], [12]. Matters are more complex in all other archaeal linages, where a family-D polymerase is additionally expressed. The biochemical properties of family-D enzymes are generally compatible with DNA replication, and it has been proposed that Pol-D copies the lagging strand, while Pol-B is involved in the leading strand synthesis [16], [17], [18], [19]. A more likely recent model suggests that in Archaea, Pol-D is responsible for replicating both DNA strands with Pol-B being used for Okazaki fragment maturation and gap filling [20]. The direct interaction of Pol-D with many key replication factors further supports its important role [21]. Genetic evidence on the roles of Pol-B and Pol-D is somewhat conflicting. Both proteins are essential for the viability of the halophilic euryarchaeon NRC-1, suggesting a key role for each in DNA replication [22]. However, in two other members of the euryarchaeal lineage, *Thermococcus kodakarensis* and *Methanococcus maripaludis*, Pol-B is not essential, inferring that Pol-D constitutes the single replicative polymerase [23], [24]. A unique property of archaeal polymerases is the inhibition of DNA extension when deaminated bases are encountered. This has been most thoroughly elucidated for Pol-B, where replication is stalled on encountering uracil or hypoxanthine in template strands [25]. X-ray structural data have indicated the presence of a pocket in the N-terminal domain that mediates tight and specific binding of both deaminated bases [26], [27]. Pol-D is slowed when uracil is present in template strands, by a mechanism that has yet to be fully characterised but appears different to Pol-B [28]. Uracil and hypoxanthine arise from hydrolytic deamination of cytosine and adenine, respectively, and result in transition mutations following replication [29]. It is assumed that the polymerase response to deaminated bases serves to suppress mutations, perhaps via replication fork regression [30], a feature that may be advantageous for organisms that live at high temperatures, which promote cytosine to uracil deamination. However, the exact function of uracil sensing is unproven, and the problem, e.g. why it is retained in mesophilic archaea but absent from thermophilic bacteria, remains [30]. Family-D polymerases are heterodimers with a small (DP1) and a large (DP2) subunit harbouring the proofreading 3′–5′ exonuclease and polymerase active sites, respectively [14], [15], [31]. DP2 shows no sequence similarity to other proteins but contains two cysteine clusters towards its C terminus, which have been tentatively suggested to form zinc fingers [15], [32]. Recently, however, similarly spaced cysteines have been observed in Eukaryotic family-B DNA polymerases and shown to serve as ligands for Zn^2 +^ and an Fe–S cluster [33], [34]. Little is known about the features of family-D polymerases, certainly much less than for all other polymerase classes. In this publication, therefore, we have sought to increase knowledge of Pol-D. Initial attempts were made to express Pol-D from *Archaeoglobus fulgidus* (Afu), *Methanopyrus kandleri*, *Methanothermobacter thermoautotrophicus*, and T*hermococcus gammatolerans* in *Escherichia coli*. Our group has previously produced *Pyrococcus furiosus* (Pfu) Pol-D in this bacterium [28]. We observed that the best quality protein, in terms of yields, purity, and solubility, was obtained from Afu and, hence, subsequent experiments were conducted with Afu Pol-D.

Alignment of the amino acid sequence of the DP2 subunit of Pol-D sequence reveals two cysteine clusters (Fig. 1). The first, farthest from the C terminus, comes in two flavours with a main consensus CX_2_CX_8_CX_2_CX_9_CX_2_CX_6-18_CX_2_C and a reduced version CX_2_CX_8_CX_2_C, which seems restricted to the *Methanococcales*, *Methanobacteriales*, *Thermoplasmatales*, *and Nanoarchaeales*. The second set, closer to the C terminus has the consensus CX_2_CX_12-14_CX_2_C. In some instances, CX_2_C is compacted to CXC, this shorter version seems unrelated to any particular Archaeal group and is distributed randomly. We have used site-directed mutagenesis to probe the role of these residues in Afu Pol-D, and three mutants, each having two closely spaced cysteines replaced by alanine, were prepared: mut2, C663A/C666A; mut4, C688A/C691A; and mut5, C1062A/C1065A (Fig. 1; for the purification of Afu Pol-D and demonstration of the lack of contaminating nucleases and the mutagenesis protocol used, see Supplementary Fig.). The activity of these mutants was investigated using a polymerase substrate comprising a Cys-5-labelled 24 base primer annealed to a 54 base template (Fig. 2a). Primer extension assay showed that mut2 and mut4 (alterations to cysteines in the cluster farthest away from the C terminus) had similar activity to the wild type (Fig. 2a). In contrast, mut5 (changes to the cysteines nearest to the C terminus) was unable to elongate the primer, even after 30 min of incubation. Analysis using inductively coupled plasma mass spectroscopy (ICP-MS) indicated that mut2 and 4, similar to the wild type, have about 1 mol of Zn per mole of protein; while in mut5, the amount of Zn present is considerably reduced (Fig. 2b). These results show that the cysteine cluster nearest the C terminal is responsible for binding a single Zn^2 +^ and, furthermore, this metal is essential for DNA polymerase activity. The role of the cysteines farthest away from the C terminus remains unclear. By analogy, with eukaryotic family-B polymerases, these thiols could serve as ligands for an Fe–S centre [33], [34]. However, Afu-Pol D, isolated from *E. coli*, shows no indication of an Fe–S cluster by UV/visible spectroscopy or analysis for inorganic sulphide (data not shown). Clearly, this cysteine cluster, and any putative metals it binds, is not required for polymerase activity. Additionally, these thiols do not play any role in the previously observed inhibition of Pol-D by template strand uracil [28]; mut2 and mut4 are slowed by uracil to a similar extent as the wild type (Fig. 2c).

Archaeal family-B polymerases are stalled by the presence of two deaminated bases, uracil and hypoxanthine, in template strands, and Pol-D is also known to be inhibited by uracil [25], [26], [27], [28]. However, any influence of hypoxanthine on Pol-D activity has yet to be tested. Using the primer extension assay described above, we have shown, for the first time, that hypoxanthine inhibits extension by Afu Pol-D (Fig. 3a). As expected from previous work, the presence of uracil also retards Afu Pol-D to a greater degree than hypoxanthine. The gels shown in Fig. 3a have been scanned to reveal the time taken for the buildup of completely extended template, the ultimate product produced by polymerase activity. With hypoxanthine, the elongation time needs to be increased some 5- to 10-fold to obtain a similar amount of final product as seen with the thymidine control; in the case of uracil, about a 20-fold increase is required. Thus, Pol-D, like Pol-B, is hindered by both uracil and hypoxanthine, with uracil being the more profound inhibitor for both enzymes. No complete high-resolution structures are known for Pol-D, so it is unclear whether the deaminated base sensing apparatus is located in DP2 or in DP1 or, indeed, if both subunits are required. It proved straightforward to purify the isolated DP2 subunit of Afu Pol-D, which showed polymerase activity, albeit at a reduced rate compared to the intact holoenzyme. With Afu Pol-D, the full-length product is visible at the first time point of 5 s (Fig. 3a), whereas Afu DP2 requires 2 min before this end product becomes visible (Fig. 3b). Previously, the DP2 subunit of Pfu Pol-D has been reported to have activity 100 times weaker than the heterodimer or no activity whatsoever [15], [35]. When the Afu DP2 subunit was used to copy a uracil-containing template, no full-length product was being seen after 20 min of incubation (Fig. 3b). With the thymidine control, Afu DP2 gave noticeable amounts of final product after 2 min and pronounced quantities after 5. With hypoxanthine, inhibition is more marginal but still apparent (compare the 2 and 5 min lanes for the thymidine control and hypoxanthine). Scanning these gels to indicate production of fully extended template makes it clear that both uracil (strongly) and hypoxanthine (more weakly) impede the passage of Afu DP2 (Fig. 3b). These results suggest that the uracil/hypoxanthine sensing apparatus is present in the large polymerase subunit. The data presented in Fig. 3a and b were repeated three times, with nearly identical results found in each instance. Previously, we described a polymerase extension assay using a mimic of the replication fork [28]. These substrates, illustrated in Fig. 3c, consist of a long hairpin template annealed to two primers, which reproduce lagging (fluorescein-labelled primer) and leading strand (cyanine 5-labelled primer) synthesis. As fluorescein and cyanine-5 display well-separated fluorescence spectra, lagging and leading strand copying can be separately monitored. These mimics were earlier used to demonstrate “*trans*” inhibition of Pfu Pol-D; the presence of uracil in the lagging template not only slows its copying but also retards the extension of the leading strand and vice versa [28]. Here, we wished to extend these studies to see if the isolated DP2 subunit retained “*trans*” inhibition ability. An initial experiment showed, as expected, that Afu Pol-D itself behaved in a similar fashion to Pfu Pol-D, and a single uracil in either the lagging or the leading template inhibits the copying of both strands (Supplementary Fig. S2). With Afu DP2, the control fork mimic, lacking any uracil, can be extended to full length in both branches, although less efficiently than observed with Afu Pol-D (Fig. 3d). As expected, polymerisation of each strand is strongly reduced when uracil is located in both branches. More interesting are the inhibition patterns observed with a single uracil in only one of the strands. Here, the gels are much more similar to those seen with the double uracil primer-template rather than the control (Fig. 3d), indicating that DP2 retains “*trans*” inhibition by uracil. Thus, the ability of a translocating Pol-D to sense the presence of uracil in a remote strand is carried by the large polymerase subunit. Again, three repeats of the data shown in Fig. 3d demonstrated very high reproducibility. It is noted that an increase in truncated products, relative to full-length product, is seen between 5 and 10 min in some cases, especially when uracil is present in the templates. Why this takes place remains unclear as the DP2 subunit lacks proofreading exonuclease activity, and the preparation, while not 100% pure, is nuclease free (Supplementary Fig. S1). Perhaps, the large Pol-D subunit and housing the polymerase active site also possess an additional, as yet uncharacterised activity that degrades deaminated base-containing sequences.

The biochemical properties of the archaeal family-D polymerase, coupled with the lethal nature of gene knockouts, have led to the conjecture that these are essential replicative polymerases [16], [20], [23], [24]. Polymerases dedicated to genome copying are expected to be highly accurate, and we have assessed the error rate of Pol-D using a plasmid-based *lacZα* gene assay, which was developed in our laboratory [36]. We have determined fidelity rates for Afu Pol-D, Afu Pol-D exo^−^ (harbouring a single amino acid substitution, H325A, in the DP1 subunit that abrogates 3′–5′ proofreading exonuclease activity), and Afu DP2. The results are given in Table 1, which shows that Afu-Pol D has an error rate of 0.24 × 10^− 5^. A previous investigation, using a PCR-based method, with Pol-D from the archaeon *Thermococcus* species 9°N reported a higher error rate of 95 × 10^− 5^ [19]. Using PCR method, it is necessary to take into account the number of template doublings when measuring fidelity, and this correction was not carried out, resulting in overestimation of the error rate [37]. We believe our value of 0.24 × 10^− 5^ is much more accurate and, as an additional control, the fidelities of Pfu Pol-B and Taq Pol were measured and found to agree with previous results, strengthening confidence in the data seen with Pol-D [37]. For comparison, the error rates of the eukaryotic, replicative polymerases ε and δ, determined using a similar *in vitro* assay, have been reported as ≤ 1.3 × 10^− 5^ and ≤ 0.2 × 10^− 5^, respectively [38]. These figures approach the detection limits of the assays but suggest that the accuracy of Pol-D, as measured *in vitro*, is compatible with it functioning as a genome copying polymerase. Using a single amino acid substitution in DP1 to disable the proofreading exonuclease increased the error rate by a factor of two. Interestingly, Afu-DP2, in which the entire DP1 subunit is absent, demonstrated the same fidelity as the H325A variant, suggesting that DP1 subunit plays a straightforward role in fidelity by simply supplying the proofreading exonuclease active site. With eukaryotic Pol δ, the proofreading exonuclease makes a similarly small contribution, although its influence is much more pronounced with Pol ε [38]. To further characterise the mistakes made by Pol-D during replication, the *lacZα* gene was completely sequenced for the 52 white (mutant) colonies observed, and the results are summarised in Supplementary Fig. S3. Although the mutations are distributed across the *lacZα* sequence, a hotspot is apparent at a run of four adenines, with 18% to 28% of the changes occurring in this region. At this site, Afu Pol-D, Afu Pol-D exo^−^, and Afu DP2 are prone to cause insertions or deletions, probably due to polymerase slippage during elongation (Supplementary Fig. S3). Base substitutions are also overrepresented at or near a run of three guanines and one run of two guanines (although not all two guanine sequences are hotspots). Base transitions are the most frequently observed change followed by frameshifts, with transversions being least abundant (Table 1). However, transversions occur more commonly with Afu Pol-D exo^−^ as compared to Afu Pol-D and Afu-DP2.

The Pol-D was first observed in the archaea in the late 1990s [13], [14], [15], but information remains sparse with relatively few publications describing the properties of these proteins. This publication furthers the knowledge of Pol-D in three areas. First, the cysteine cluster nearest the C terminal of the DP2 subunit is shown to coordinate Zn^2 +^ and to be absolutely essential for polymerase activity. Second, Pol-D is demonstrated to be inhibited by the hypoxanthine, extending earlier studies that indicated inhibition by uracil. The uracil/hypoxanthine-sensing region is localised to the DP2 subunit and shown to act both in *cis* (i.e., deaminated bases inhibit replication of the strand on which it is located) and in *trans* (i.e., deaminated bases on one strand of a replication fork inhibit copying of the other strand). *Trans*-inhibition is never seen with Pol-B, where deaminated bases stall replication only at the strand on which they are located, emphasising a fundamental difference in the mechanism by which Pol-B and Pol-D respond to the uracil/hypoxanthine. Third, the fidelity of Pol-D has been accurately determined and shown to be compatible with a genome copying enzyme. The proofreading exonuclease activity has only a modest, about twofold, influence on accuracy. Probably, the outstanding question concerns the function of the cysteine cluster away for the C terminus. These amino acids are not required for polymerase activity or deaminated base sensing. It may be that these cysteines support an Fe–S cluster, which, in the recent years, have been demonstrated in many nucleic acid processing enzymes such as glycosylases, primases, helicases, nucleases, transcription factors, RNA polymerases, and RNA methyltransferases [39]. Furthermore, the cysteine arrangement in archaeal Pol-D is reminiscent of that observed in eukaryotic family-B polymerases, the latter having been identified as Fe–S and Zn-binding proteins [33], [34]. All attempts to observe an Fe–S cluster in Afu Pol-D, prepared from *E. coli* under aerobic conditions, proved negative; and UV/visible spectroscopy, ICP-MS, and inorganic sulphide analysis gave no indication for the presence of such a system (data not shown). Attempted expression under anaerobic conditions, with iron and sulphur supplementation and using *E. coli* strains enhanced for Fe–S biosynthesis [40], [41], [42], all proved negative, as did the expression of Pol-D from a number of other archaea. If Pol-D does possess an Fe–S cluster, it seems clear that it is not essential for enzymatic activity and cannot be produced using heterologous expression in *E. coli*. This is also the case with eukaryotic polymerases, where assembly of the Fe–S centre requires expression yeast rather than *E. coli* [33], [34]. Interestingly, the Fe–S cluster in eukaryotic polymerase is also not required for activity but rather mediates protein–protein interactions and holoenzyme formation, an observation compatible with the nonessential nature of the unassigned archaeal thiol cluster. The absence of a putative Fe–S cluster might also underlie the difficulties very commonly observed in obtaining highly homogenous preparations of Pol-D, as seen here and also by others [15], [16], [20], [31]. Therefore, it is likely that the full resolution of the metal content of Pol-D and purification to the standard required for X-ray crystallography structural analysis will require overexpression in an archaeal host, and such experiments are currently under consideration.

## Acknowledgements

This work was supported by the UK BBSRC (grant number: BB/K005359/1). We thank Dr. Patrik Jones (University of Turku, Finland) for *E. coli* BL21(DE3)∆* iscR* and Professor Dennis Dean (Virginia Tech University, USA) for *E. coli* BL21(DE3) (pDB1282).

## Figures and Tables

**Fig. 1:**
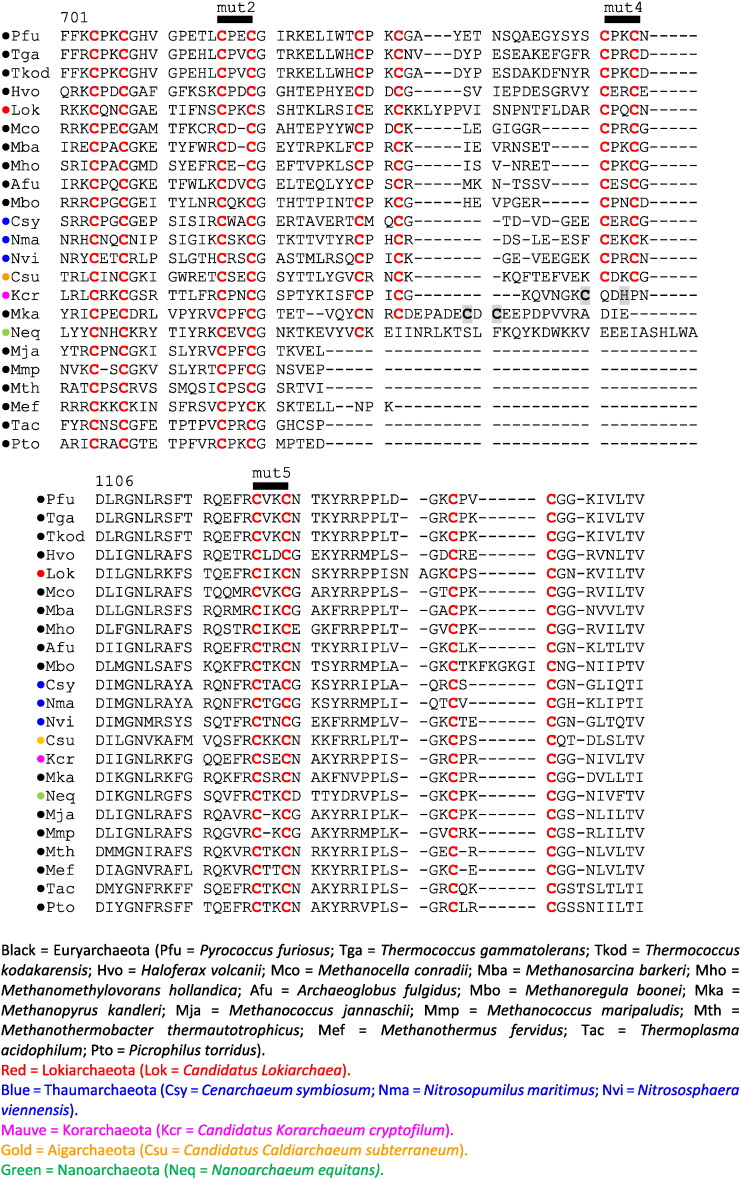
Amino acid sequence alignment showing conservation of cysteines in the C-terminal region of the DP2 (polymerase) subunit of DNA Pol-D. The number (701) above the alignment refers to the residue position in the Pfu protein. The names of the archaeal species used in the alignment are given in a three letter code, and the coloured dots refer to their phylogenetic group. Conserved cysteines are highlighted in red, alternative cysteines and, in one case, a histidine are shaded in grey. The more N-terminal cysteines occur either as an extended motif of eight cysteines (Pfu type) or a compact four cysteine cluster (Mja type). The more C-terminal motif is more regular with four cysteines. The black bars above the alignment show the pairs of cysteines mutated to alanine in Afu Pol-D. Below the lineup is a key showing the correspondence between the full name of the archaea and the three letter abbreviation used and the phylogenetic group of the organisms analysed. Pol-D DP2 amino acid sequences were downloaded from the National Centre for Biotechnology Information (http://www.ncbi.nlm.nih.gov/), and alignments were generated using Multalin (http://multalin.toulouse.inra.fr/multalin/) [43].

**Fig. 2:**
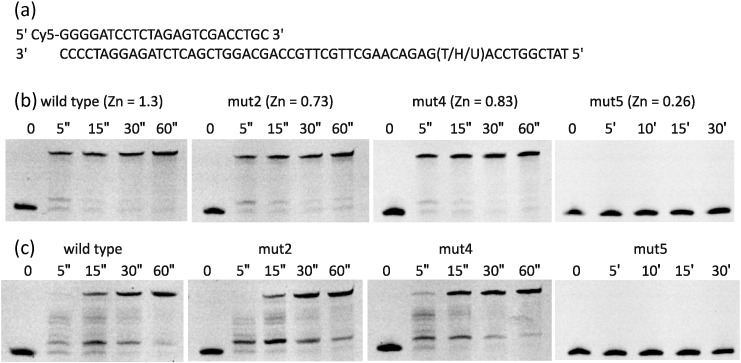
The influence of cysteine deletion mutants on the polymerase activity of Afu Pol-D. (a) The primer-templates used for assessing polymerase activity. The template contains thymidine, hypoxanthine, or uracil at the + 23 position. (b) Extension of the control (thymidine-containing) primer-template by wild-type Afu Pol-D and mut2, mut4, or mut5 (Fig. 1) for the times indicated in seconds (″) or minutes (′). The mole fraction of Zn, determined by ICP-MS, present in each protein is also given here. (c) Extension of the uracil-containing primer-template by the Pol-D variants. Polymerase activity was measured in 120 μl of 10 mM Tris–HCl (pH 9), 50 mM KCl, 10 mM MgCl_2_, 10 mM DTT, and 200 μM each of dNTPs with 20 nM of the DNA substrate and 80 nM of polymerase. The solution was incubated at 50 °C for the times shown, and 20 μl aliquots were then quenched by addition to an equal volume of 95% formamide, 10 mM EDTA, and 2 μM of a “competitor” oligodeoxynucleotide [44]. Samples were heated for 10 min at 95 °C, transferred to ice, and centrifuged for 2 min at 13,000 rpm. Analysis was by denaturing PAGE (17% polyacrylamide, 8 M urea), and extension products were visualised with a Typhoon 9500 (GE Healthcare Life Sciences) and analysed using Image Quant software. For determination of ZN content using ICP-MS, Afu Pol-D samples (18 μM to 30 μM) were buffer exchanged into 50 mM Hepes–NaOH (pH 7.5), 100 mM NaCl, 1 mM EDTA, and 1 mM DTT. Triplicated aliquots (75–150 μl) were diluted into 2% Suprapur HNO_3_ (Merck) to a final volume of 3 ml and analysed by ICP-MS (Thermo Scientific, X-Series) operating in collision cell mode (3.0 ml min^− 1^ 8% H_2_ in He). Isotope ^66^Zn was monitored 100 times using the peak-jump method (40 ms integration time on each of seven channels, separated by 0.02 atomic mass units), read in triplicate for each sample. The sample results were compared with matrix-matched elemental standards to calculate metal concentrations. For the preparation, purity, and mutagenesis of Afu Pol-D, see Supplementary Fig. S1.

**Fig. 3:**
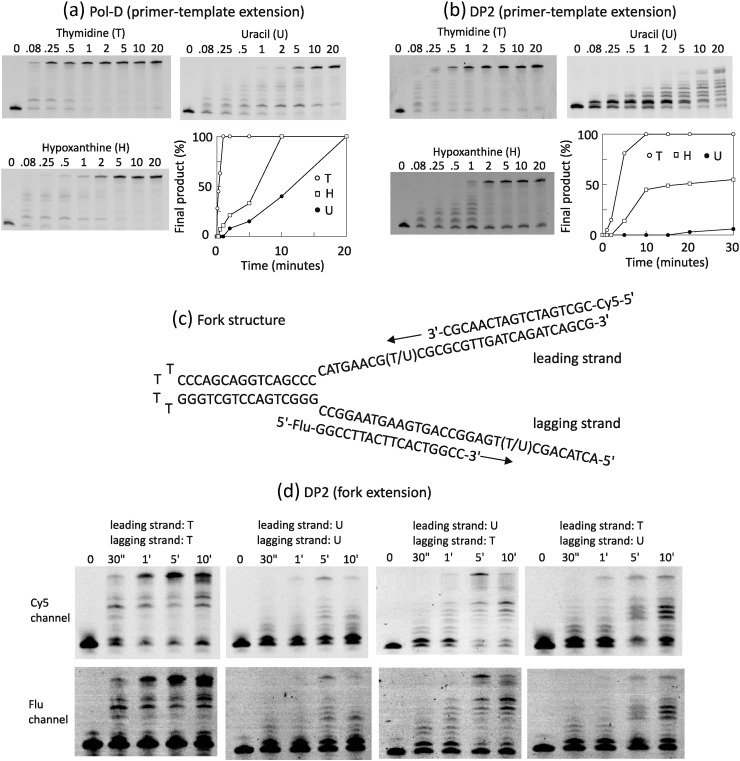
Inhibition of Afu Pol-D and Afu DP2 by the presence of hypoxanthine and uracil in primer-templates and replication fork mimics. (a) Extension of the primer-template (see Fig. 2 for the sequences used) with thymidine, hypoxanthine, or uracil at position + 23 by Afu Pol-D for the times indicated in seconds (″) or minutes (′). (b) As in Fig. 3A but with Afu DP2 used in place of Afu Pol-D. The graphs indicate the amount of the final product (i.e., the fully extended primer) produced at increasing times. (c) Sequence and structure of the primer-template fork mimic. A long hairpin oligodeoxynucleotide constitutes the backbone of the fork. Annealing of the Cy5 (cyanine-5)- and Flu (fluorescein)-labelled primers results, respectively, in leading and lagging strand branches. Both branches have a solitary uracil (thymidine in controls) four bases ahead of the primer-template junction. (d) Extension of the fork mimics with the uracil/thymidine-leading/lagging strand combinations shown above the gel panels. Both strands are simultaneously extended in the same experiment but can be individually monitored thanks to the spectral separation of the Cy5 and Flu dyes. The top gel in each pair (Cy5 channel) shows leading strand extension and the bottom gel (Flu channel) copying of the lagging strand. The extension times used are given in seconds (″) and minutes (′). The assay conditions and analysis were identical to those given in the legend to Fig. 2.

**Table 1:** The fidelity of Afu Pol-D and reference polymerases using the pSJ3 assay

Polymerase	Total number of colonies	White (mutant) colonies	Error Rate^a^	Transversions^b^ (%)	Transitions^b^ (%)	Frameshifts^b^ (%)
Taq Pol	18,959	17	0.59 × 10^− 5^	nd	nd	nd
Pfu Pol B	17,971	3	0.09 × 10^− 5^	nd	nd	nd
Afu Pol-D	28,473	11	0.24 × 10^− 5^	9	64	27
Afu Pol-D exo^−^ (H325A)	18,114	14	0.51 × 10^− 5^	28.5	43	28.5
Afu DP2	34,834	27	0.51 × 10^− 5^	15	55	30

Polymerase fidelity was determined using the pSJ3 plasmid-based *lacZ*α reporter gene assay [36]. A typical pSJ3 gap-filling reaction was carried out in 20 μl of 10 mM Tris–HCl (pH 9), 50 mM KCl, 10 mM MgCl_2_, and 10 mM DTT containing 40 ng of gapped plasmid, 1 mM each dNTP, and 50 nM of the polymerase. The reaction was incubated 30 min at 70 °C. A small sample was tested using *Eco*RI (New England Biolabs) digestion followed by 1% agarose electrophoresis to confirm successful extension. The determination of fidelity by ultimately scoring ratios of blue/white colonies and the sequencing of mutant (white) colonies has been described [36].

a The calculation of error rates was as described previously [36].

b Assignment of transversions, transitions, and frameshifts was based on the sequencing data given in Supplementary Fig. S3.

## Appendix A Supplementary Data

Supplementary Fig. S1: the purification, purity, and mutagenesis of *Archaeoglobus fulgidus* DNA polymerase D Pol-D, lack of nuclease contamination in Pol-D. Supplementary Fig. S2: *trans*-inhibition of Afu Pol-D by uracil. Supplementary Fig. S3: the mutation spectrum of Afu Pol-D. Image 2
