# Supplementary material for: Archaeoglobus Fulgidus DNA Polymerase D: A Zinc-Binding Protein Inhibited by Hypoxanthine and Uracil
Source: J Mol Biol. 2016 Jul 17;428(14):2805–13. doi: 10.1016/j.jmb.2016.06.008 (PMC4942837; doi:10.1016/j.jmb.2016.06.008)
Supplement: Supplementary file 1 — Supplementary Fig. S1: the purification, purity, and mutagenesis of Archaeoglobus fulgidus DNA polymerase D Pol-D, lack of nuclease contamination in Pol-D. Supplementary Fig. S2: trans-inhibition of Afu Pol-D by uracil. Supplementary Fig. S3: the mutation spectrum of Afu Pol-D. [file mmc1.pdf]

Supplementary figure S1

Purification and characterisation of *Archaeoglubus fulgidus* Pol-D variants

Archaeoglobus fulgidus genomic DNA was obtained from DSMZ (The German Collection of Microorganisms and Cell Cultures, Braunschweig, Germany). The genes encoding the DP1 and DP2 sub-units of DNA polymerase-D were amplified by the PCR using Velocity DNA polymerase (Bioline, London, UK). The primers used were: DP1 upstream, GATCCATATGGTAATTAAAAATATCGATGCC (adds NdeI site, underlined); DP1 downstream, GATCGCGGCCGCTTACTCACCGTAGAACCTCAG (adds NotI site, underlined); DP2 upstream, AAAACATATGGATGCAACTCTTGACAG (adds NdeI site, underlined); DP2 downstream, AAAAGGATCCTCAAACGAAATCGGATATTG, (adds BamHI site, underlined). The PCR products were purified (QIAquick PCR purification kit, QIAGEN, Manchester, UK) and digested with the appropriated restriction enzyme (New England Biolabs, Hitchin, UK). The DP1 gene was inserted into pET24a (Km^R) between the NdeI/NotI sites to give pJAR035 and the DP2 gene into pET21a (Amp^R) between the NdeI/BamHI sites to give pJAR076 using T4 DNA ligase (New England Biolabs). The inserted genes were completely sequenced (GATC Biotech, Cambridge, UK), which showed no errors were present. The PCR overlap extension method was employed for site-directed mutagenesis [1]. The following mutants were prepared:

1) H325A (DP1 sub-unit), a 3’-5’ proof reading exonuclease deficient version (starting plasmid, pJAR035, primers CTCTCCCGGAAACGCTGATGCGGTCAGG/CCTGACCGCATCAGCGTTTCCGGGAGAG, mutant plasmid pJAR082)
2) C663A/C666A (DP2 sub-unit), mut2 lacking two cysteines (starting plasmid pJAR076, primers CTGGCTCAAGGCCGACGTTGCTGGTGAGCTTACAGAG/TAAGCTCACCAGCAACGTCGGCCTTGAGCCAGAA, mutant plasmid pJAR092).
3) C688A/C691A (DP2 sub-unit), mut4 lacking two cysteines (starting plasmid pJAR076, primers, CTCAAGCGTCGCCGAGAGCGCTGGCAGAGAGTGTGAG/TCTCTGCCAGCGCTCTCGGCGACGCTTGAGGTGTTC, mutant plasmid pJAR093).
4) C1062A/C1065A (DP2 sub-unit), mut5 lacking two cysteines (starting plasmid pJAR076, primers, GGAGTTCCGCGCTACAAGGGCCAACACGAAGTACCGC/ACTTCGTGTTGGCCCTTGTAGCGCGGAACTCCTGTCTTG, mutant plasmid pJAR094).

All Afu Pol-D derivatives (wild type, mutants and DP2 sub-unit) were overexpressed in *Escherichia coli* BL21(DE3) pLysS harbouring the appropriate Pol-D encoding plasmid(s). Cells were grown at 37 °C in terrific broth supplemented with 3g/l NaCl and the appropriate antibiotic(s) to an A_{600} of 0.8. Protein expression was induced by addition of 1mM IPTG followed by growth for a further 5 hours and cells pelleted and stored at -80 °C until needed. Cells were suspended in 50 mM Hepes-NaOH (pH 7.5), 1 mM EDTA, 1 mM DTT, 150 mM NaCl, 10 % (v/v) glycerol and EDTA-free protease inhibitor (Roche) and lysed by sonication (on ice). DNase1 (Roche) treatment (0.1 µg/ml) for 30 minutes at 37 °C was used to degrade DNA, followed by a heat step (75 °C for 20 minutes) to inactivate DNase1 and denature most host proteins. Cell debris and precipitated proteins were pelleted by centrifugation and the supernatant was filtered (Millex-HA 0.45 µM) prior to application to a HiTrap™ 5-ml Heparin HP (GE Healthcare) column equilibrated with the above buffer lacking the protease inhibitor. The column was washed thoroughly with this buffer and the target protein eluted using a 30 ml linear gradient of 150-1000 mM NaCl. The eluate was analysed by 10 % SDS PAGE and appropriate fractions pooled and stored in small aliquots at -80 °C. The identity of the proteins was confirmed by mass spectrometry (Proteomic service, York University, UK).

[1] S.N. Ho, H.D. Hunt, R.M. Horton, J.K. Pullen, L.R. Pease, Site-directed mutagenesis by overlap extension using the polymerase chain reaction, Gene 77 (1989) 51–59.

Supplementary figure 1 (continued)

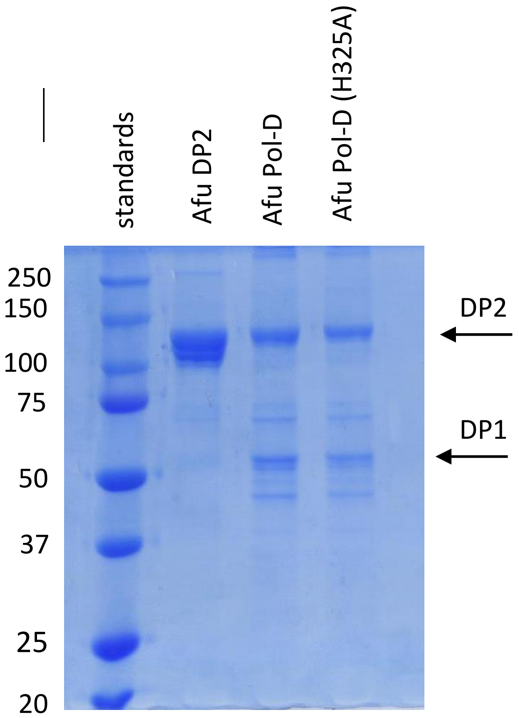

Purity of *Archaeoglobus fulgidus* DP2, Pol-D(wild-type) and Pol-D (H325A) assessed by denaturing SDS-PAGE with Coomassie Blue staining. The proteins are largely pure and lower molecular weight contaminants score as Pol-D by mass spectroscopy and arise by proteolytic cleavage. Further purification by gel filtration was not possible, suggesting that the proteolytic fragments remain assembled under native conditions and only become apparent on denaturation. The purity of the thiol mutants mut2, mut4 and mut5 were similar (data not shown). The molecular masses of the standards (kDa) are given.

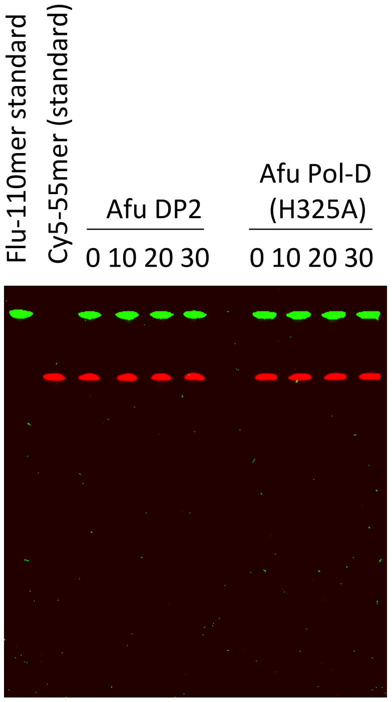

Nuclease contamination check. The presence of contaminating nucleases in Pol-D was checked using a primer-template with dual fluorophores (primer 5′-labelled with cyanine5, template labelled with fluorescein, mixed in a 1:1 ratio). The primer-template (20 nM) was incubated, at 50 °C for up to 30 minutes, with Afu DP2 or Afu Pol-D(H325A) in 120 μl of 10 mM Tris-HCl pH 9, 50 mM KCl, 10 mM $MgCl_2$, 10 mM DTT. 20 μl aliquots were quenched by addition of an equal volume of 95 % formamide, 10 mM EDTA and the samples heated for 10 minutes at 95 °C, transferred to ice and centrifuge for 2 minutes at 13,000 rpm. Analysis was by denaturing PAGE (17 % acrylamide, 8 M urea, gel run at 60 °C). Degradation was visualized with a Typhoon 9500, set for dual imaging of cyanine5 (red channel) and fluorescein (green channel) and analysed using Image Quant. This assay was not performed with wild type Pol-D due to expected degradation by the 3’-5’ proof-reading exonuclease activity.

Primer-template used in the nuclease contamination check:

5’FluCCCAGCAGGTCAGTCATTCGGATAACTGGGTTTAACTAGACTAGAGAAGGATAGGTCCTGCAATTCTGGCATGACTCGAATCTCGTAACGTTACTTACCATTGCAGTGGG 3’
3'AGGACGTTAAGACCGTACTGAGCTTAGAGCATTGCAATGAATGGTAACGTCACCC Cy55'

Supplementary figure S2: Trans inhibition of Afu Pol-D by uracil

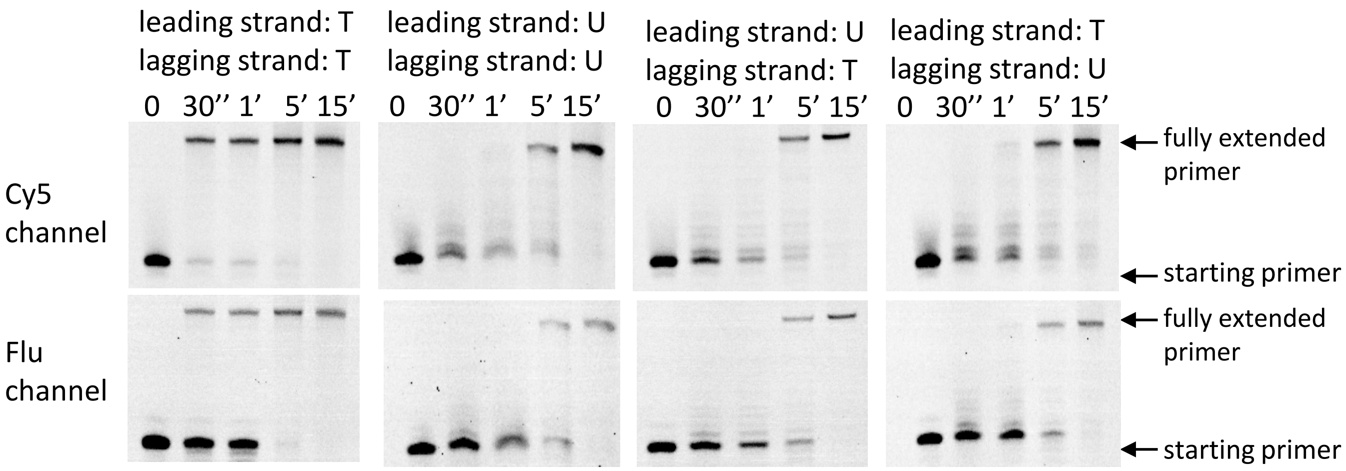

Supplementary figure S2. “Trans” inhibition of Afu Pol-D by uracil. Extension of the fork mimics with the uracil/thymidine-leading/lagging strand combinations shown above the gel panels. For a schematic of the fork mimic with definition of the leading and lagging strands see Figure 3 in the main paper. Both strands are simultaneously extended in the same experiment but can be individually monitored thanks to the spectral separation of theCy5 and Flu dyes. The top gel in each pair (Cy5 channel) shows leading strand extension and the bottom gel (Flu channel) copying of the lagging strand. The extension times used are given in seconds (″) and minutes (′). As discussed in the text the presence of uracil inhibits the polymerase both in “cis” (uracil and polymerase on the same strand) and in “trans” (uracil and polymerases on different strands). For a comparison with Afu DP2 see main paper Figure 3.

Supplementary figure S3: Mutation spectra of Afu Pol-D variants

Afu Pol-D wild type

```
                                                       T
TCAGCT ATG ACC ATG ATT ACG AAT TCA CTG GCC GTC GTT TTA CAA CGT CGT
     A
GAC TGG GAA AAC CCT GGC GTT ACC CAA CTT AAT CGC CTT GCA GCA CAT CCC
          2x      A                        T
                AA  T                      T
CCT TTC GCC AGC TGG CGT AAT AGC GAA GAG GCC CGC ACC GGT CGC CCT TCC

CAA CAG TTC C
```

Afu Pol-D exo⁻ (H325A)

```
TCAGCT ATG ACC ATG ATT ACG AAT TCA CTG GCC GTC GTT TTA CAA CGT CGT
                                                             C
    CAT                          T   CC                      C
GAC TGG GAA AAC CCT GGC GTT ACC CAA CTT AAT CGC CTT GCA GCA CAT CCC
         3x
                 AA
CCT TTC GCC AGC TGG CGT AAT AGC GAA GAG GCC CGC ACC GGT CGC CCT TCC

CAA CAG TTC C
```

Afu DP2

```
                                    C
                        T    G      G
TCAGCT ATG ACC ATG ATT ACG AAT TCA CTG GCC GTC GTT TTA CAA CGT CGT
     A                                       A
A    AA          T                G           A              AC
GAC TGG GAA AAC CCT GGC GTT ACC CAA CTT AAT CGC CTT GCA GCA CAT CCC
         6x
                 AA
                CAA
CCT TTC GCC AGC TGG CGT AAT AGC GAA GAG GCC CGC ACC GGT CGC CCT TCC

CAA CAG TTC C
```

Supplementary figure S3. Mutations detected by DNA sequencing, in all the white colonies produced by the Afu-polymerase variants using the plasmid-based fidelity assay. The locations underlined in light represent single base deletions; the heavily underlined locations indicate single base insertions. The letters above the sequence show single base substitutions. A number followed by x means that that particular mutation was found in the number of colonies indicated. The four adenine hotspot and the runs of guanines that are frequently mutated are shown in red.
